# Cardiovascular magnetic resonance measures of aortic stiffness in asymptomatic patients with type 2 diabetes: association with glycaemic control and clinical outcomes

**DOI:** 10.1186/s12933-018-0681-4

**Published:** 2018-03-05

**Authors:** Peter P. Swoboda, Bara Erhayiem, Rachel Kan, Adam K. McDiarmid, Pankaj Garg, Tarique A. Musa, Laura E. Dobson, Klaus K. Witte, Mark T. Kearney, Julian H. Barth, Ramzi Ajjan, John P. Greenwood, Sven Plein

**Affiliations:** 1 Multidisciplinary Cardiovascular Research Centre & Division of Biomedical Imaging, Leeds Institute of Cardiovascular and Metabolic Medicine, LIGHT Laboratories, Clarendon Way, University of Leeds, Leeds, LS2 9JT UK; 2 Leeds Teaching Hospitals NHS Trust, Great George Street, Leeds, UK

**Keywords:** Cardiovascular magnetic resonance, Aortic distensibility, Pulse wave velocity, Cardiovascular risk, Renin–angiotensin–aldosterone

## Abstract

**Background:**

We aimed to investigate in patients with type 2 diabetes whether aortic stiffness is: (i) associated with glycaemic control, (ii) associated with adverse outcomes and (iii) can be reversed on treatment with RAAS inhibition.

**Methods:**

Patients with type 2 diabetes (N = 94) and low vascular risk underwent assessment of cardiovascular risk and CMR assessment of ascending aortic distensibility (AAD), descending aortic distensibility (DAD) and aortic pulse wave velocity (PWV). Of these patients a subgroup with recent onset microalbuminuria (N = 25) were treated with renin–angiotensin–aldosterone system (RAAS) inhibition and imaging repeated after 1 year. All 94 patients were followed up for 2.4 years for major adverse cardiovascular disease (CVD) events including myocardial infarction detected on late gadolinium enhancement CMR.

**Results:**

Ascending aortic distensibility, DAD and PWV all had a significant association with age and 24 h systolic blood pressure but only AAD had a significant association with glycaemic control, measured as HbA1c (Beta − 0.016, P = 0.04). The association between HbA1c and AAD persisted even after correction for age and hypertension. CVD events occurred in 19/94 patients. AAD, but not DAD or PWV, was associated with CVD events (hazard ratio 0.49, 95% confidence interval 0.25–0.95, P = 0.01). On treatment with RAAS inhibition, AAD, but not DAD or PWV, showed significant improvement from 1.51 ± 1.15 to 1.97 ± 1.07 10^−3^ mmHg^−1^, P = 0.007.

**Conclusions:**

Ascending aortic distensibility measured by CMR is independently associated with poor glycaemic control and adverse cardiovascular events. Furthermore it may be reversible on treatment with RAAS inhibition. AAD is a promising marker of cardiovascular risk in asymptomatic patients with type 2 diabetes and has potential use as a surrogate cardiovascular endpoint in studies of novel hypoglycaemic agents.

*Clinical trials registration* https://clinicaltrials.gov/ct2/show/NCT01970319

## Introduction

With aging there is progressive stiffening of the aorta that appears to be accelerated by the presence of additional risk factors such as hypertension and diabetes [1]. Aortic stiffness can be assessed by cardiovascular magnetic resonance (CMR) either directly as aortic distensibility (AD) the relative change in aortic cross sectional area divide by pulse pressure; or indirectly as pulse wave velocity (PWV) the propagation speed of the velocity wave between two aortic locations. PWV is proportional to the square root AD by the Bramwell-Hill equation [2]. These techniques do not expose the patient to ionising radiation or contrast agent and can image three dimensional aortic characteristics at any point along the vessel [3].

Increased aortic stiffness can be detected in patients with type 2 diabetes with and without established cardiovascular disease by CMR [4–6] and applanation tonometry [7–10]. Although CMR measures of aortic stiffness have been shown to predict vascular morbidity [11, 12] studies specific to the diabetic population have not yet been conducted.

Patients with diabetes and microalbuminuria have even further elevated cardiovascular risk [13, 14]. Data from clinical trials suggests that the use of renin–angiotensin–aldosterone system (RAAS) inhibition in this patient group may reduce this risk [15, 16]. It has been shown that AD and PWV measured by CMR can be improved by RAAS inhibition in other high risk disease cohorts [17, 18] but this principle is not yet been tested in diabetes.

We aimed to investigate in patients with type 2 diabetes whether aortic stiffness is: (i) associated with glycaemic control, (ii) associated with adverse outcomes and (iii) can be reversed on treatment with RAAS inhibition. We also aimed to compare three CMR measures of aortic stiffness including ascending aortic distensibility (AAD), descending aortic distensibility (DAD) and aortic pulse wave velocity (PWV).

## Methods

**Fig. 1:**
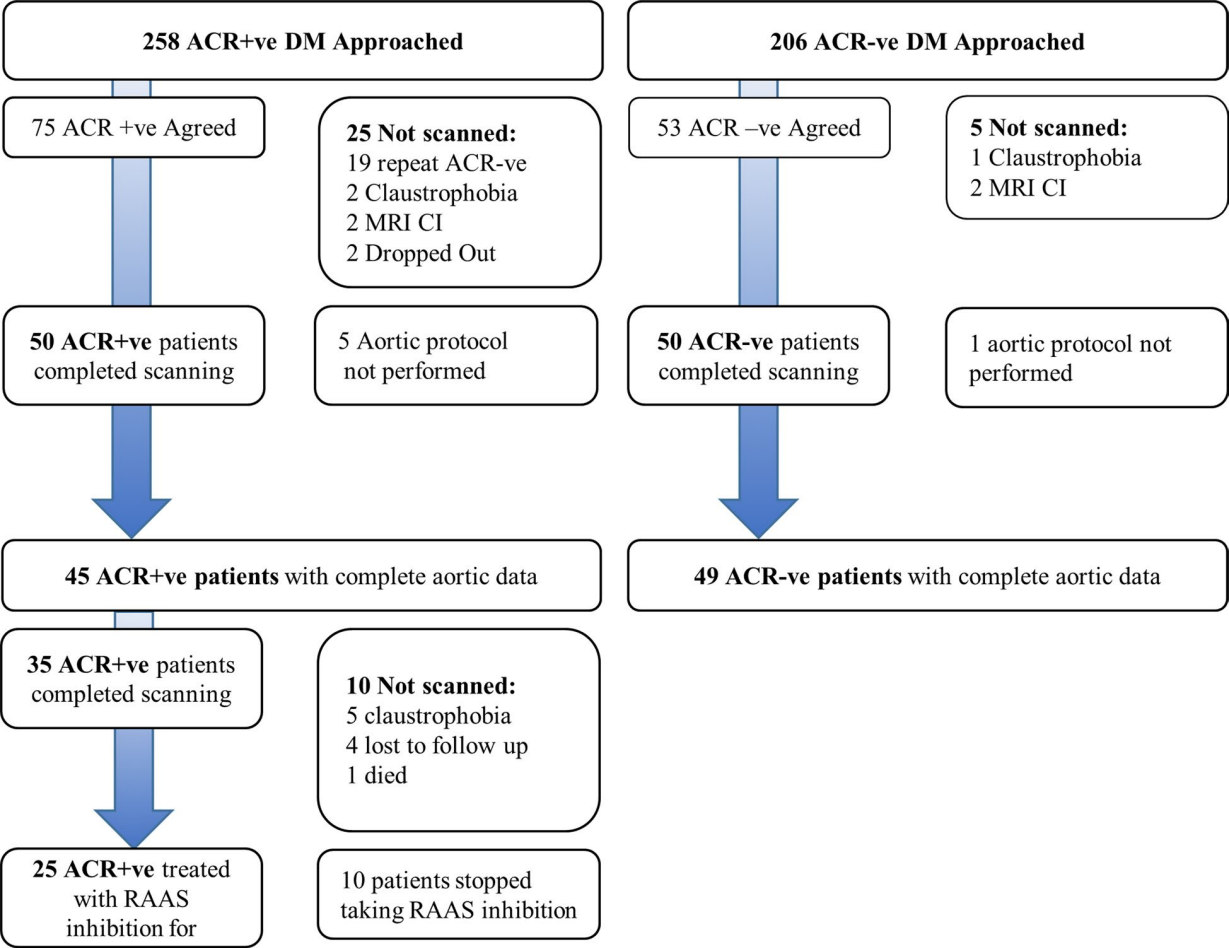
Flow chart of patient recruitment

We measured aortic stiffness by CMR in 94 asymptomatic patients with type 2 diabetes from a cohort of 100 patients with type 2 diabetes studied to investigate the relationship between microalbuminuria, cardiac remodelling and fibrosis (Fig. 1) [19, 20]. Patients were recruited from 30 primary care health centres in the local area between August 2013 and March 2015 [20]. Exclusion criteria for all subjects were known cardiac disease, kidney disease (eGFR < 30 ml/min/1.73 m^2^), uncontrolled hypertension, treatment with insulin or angiotensin converting enzyme (ACE) inhibitor/angiotensin receptor blocker (ARB). All patients underwent 24 h blood pressure (BP) monitoring with a Welch-Allyn 6100 ambulatory blood pressure monitor. All HbA1c measurements since diagnosis were recorded from review of records.

We specifically recruited patients with persistent microalbuminuria (N = 45) who were due to be started on an ACE inhibitor by their primary care team following the baseline investigations [21]. ACE inhibitors were uptitrated to maximum tolerated dose and those who could not tolerate an ACE inhibitor because of cough were changed to an ARB. All testing was repeated after 1 year treatment with RAAS inhibition. In addition 20 age and sex matched healthy controls underwent identical CMR studies.

The study was approved by the National Research Ethics Service (13/YH/0098) and conducted in accordance with the declaration of Helsinki. All subjects gave informed written consent.

### CMR protocol

Patients and controls underwent CMR using an identical protocol on a dedicated cardiovascular 3 Tesla Philips Achieva system equipped with a 32 channel coil and MultiTransmit^®^ technology.

**Fig. 2:**
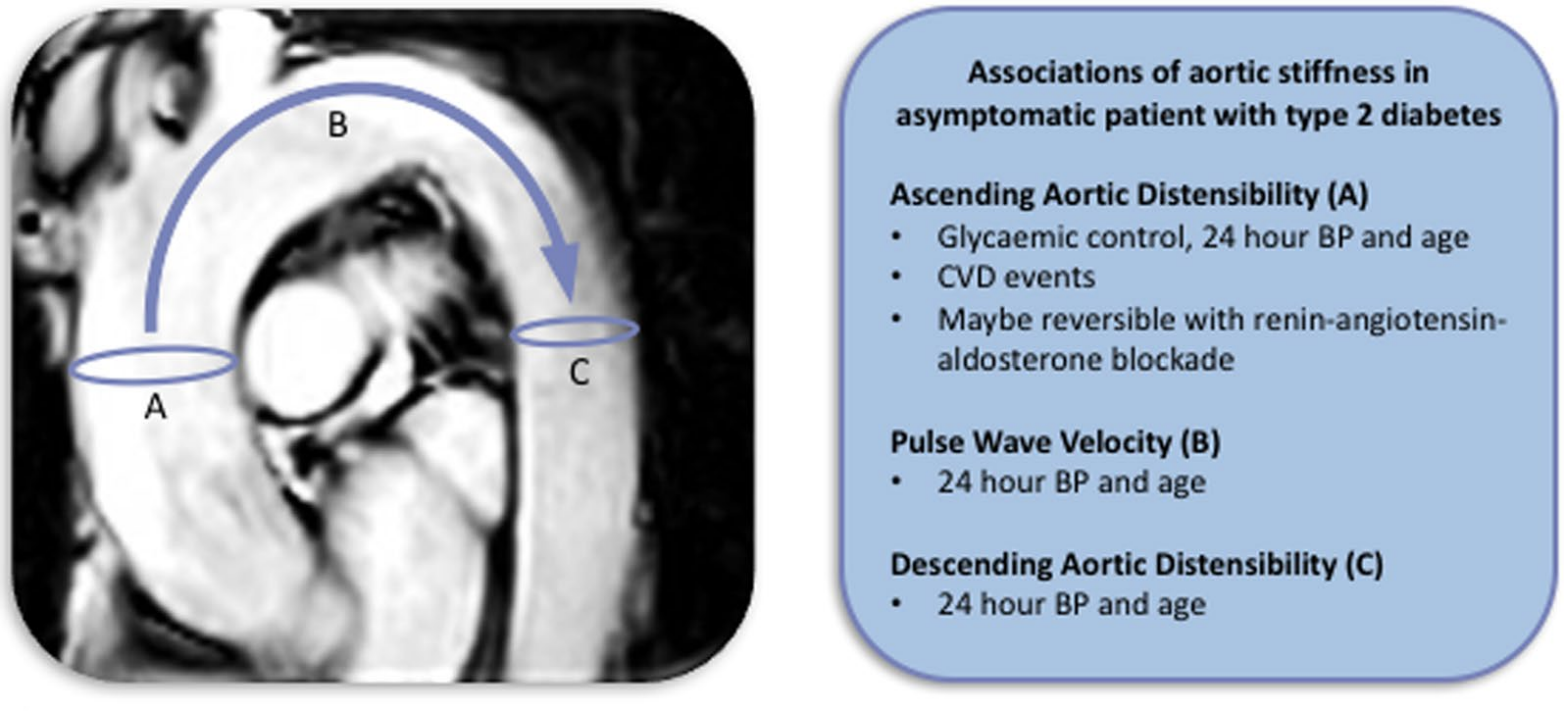
Associations of three measures of aortic stiffness in asymptomatic patients with type 2 diabetes

For aortic distensibility, brachial artery blood pressure was recorded by Dinamap (Critikon, Tampa, USA) immediately prior to high temporal resolution multi-phase SSFP cine imaging (retrospective gating, slice thickness 10 mm, acquired spatial resolution 1.2 × 1.2 mm, acquired temporal resolution 50 phases, repetition time 2.6 ms, echo time 1.3 ms, breath-held, acquired transverse to the ascending and descending thoracic aorta at the level of the pulmonary artery bifurcation) (Fig. 2) [4]. Aortic PWV was assessed using identical geometric planning with retrospectively gated, through-plane, phase-contrast velocity encoded images (single slice, 10 mm thick, acquired spatial resolution 2.9 × 2.9 mm, acquired temporal resolution 50 phases, repetition time 4.7 ms, echo time 2.8 ms, typical FOV 320, and VENC 200 cm/s, breath-held).

Late gadolinium enhancement (LGE) imaging of the heart was carried out more than 6 min after contrast injection (0.15 mmol/kg Gadovist, Bayer Pharma) using inversion recovery-prepared T1-weighted echo. The optimal inversion time to null signal from normal myocardium was determined using a Look-Locker approach (acquired spatial resolution 1.54 × 1.76 mm, TR 3.5 ms, TE 2.0 ms, flip angle 25^°^, breath-held) performed in 10–12 short axis slices with further slices acquired in the vertical and horizontal long axis orientations, phase-swapped or imaged in systole, if indicated based on LGE imaging obtained or wall-motion abnormality.

### CMR interpretation

Cardiovascular magnetic resonance data were assessed quantitatively using commercially available software (CVI42, Circle Cardiovascular Imaging Inc. Calgary, Canada) blinded to glycaemic status. To derive the aortic distensibility of the ascending and descending thoracic aorta, cross sectional measurements were made by manual planimetry of the endovascular-blood pool interface for each phase to determine the maximal and minimal aortic dimensions. Aortic distensibility (mmHg^−1^) was calculated using the equation:

$${\text{Aortic distensibility}}\, = \,\Delta {\text{ aortic area}}/({\text{pulse pressure}}\, \times \,{\text{minimum aortic area}}).$$

Aortic PWV (m/s) was calculated by dividing the distance separating two locations and the transit time needed to cover this distance [22]. Analysis was performed using a validated software (PMI v0.4, https://github.com/plaresmedima/PMI-0.4-Runtime-CMRLeeds) based on IDL 6.4 (ITT Visual Information Systems, Boulder, USA) [23]. The distance between the ascending and descending aorta was measured manually from the sagittal/oblique cines of the aortic arch. Transit time was calculated using the foot–foot delay method from velocity encoded images of the ascending and descending aorta, manually contoured to derive velocity–time curves [24].

The presence of silent MI was identified by two physicians experienced (5 and 15 years) in CMR interpretation based upon typical subendocardial distribution of LGE present.

### Laboratory methods

Cholesterol, hsCRP and microalbumin were measured on Siemens Advia (Siemens Healthcare Diagnostics, Camberley, UK) with typical coefficient of variability (CV) 0.94, 3.7 and 2.2%, respectively. HbA1c was measured on Tosoh G8 (Tosoh Bioscience, Tessenderlo, Belgium) with typical CV 1.4%. Serum aldosterone was measured in the SAS Steroid Centre (Leeds Teaching Hospitals) with an in-house radio-immunoassay with typical CV 11% at 218 pmol/L.

### Follow up

Patients were followed up by review of electronic and clinical records for CVD events including cardiovascular death, myocardial infarction (either silent detected on LGE CMR or clinically recognised during the follow up period), stroke, heart failure or arrhythmia.

### Statistical analysis

Statistical analysis was performed using IBM SPSS^®^ Statistics 22.0 (IBM Corp., Armonk, NY). Continuous variables were expressed as mean ± SD. Categorical variables were expressed as N (%). Logistic regression was performed to identify clinical and aortic parameters associated with CVD events over follow up. Univariable linear regression was performed to identify associations between clinical parameters and AAD, DAD and PWV. Only factors with a significant association on univariable regression (P < 0.05) were included in multivariable linear regression. In those who underwent treatment with RAAS inhibition paired t tests were used to compare parameters before and after treatment. When normally distributed, data are presented as mean ± SD. P < 0.05 was considered statistically significant.

## Results

Ninety-four patients had a CMR protocol that included aortic imaging and were included in this study. Patients had a mean age of 61 ± 11 years (range 32–86), Table 1. 81% of participants were male with mean duration of diabetes of 5 ± 5 years and HbA1c of 62 ± 16 mmol/mol. The majority were on metformin therapy (88%) with 34% receiving, a sulphonylurea 34, 10% a gliptin and only 4% took another hypoglycaemic agent (exanatide, pioglitazone, dapagliflozin and repaglinide, in one patient each). On 24 h ambulatory monitoring, blood pressure was well controlled at 131 ± 15/72 ± 9 mmHg. Only 13% of patients were taking an antihypertensive which included a calcium channel blocker 11%, a diuretic 4% and a beta blocker 3%. Twenty age and sex matched healthy controls were recruited with 7 (70%) male, mean age 56 ± 11, clinic blood pressure 127 ± 10/77 ± 9 mmHg and HbA1c of 38 ± 3 mmol/mol.

**Table 1:** Baseline Characteristics presented as mean ± standard deviation for continuous or N (%) for categorical data

	Diabetes mellitus	Control	P value
N	94	20	
Age (years)	60.8 ± 11.1	57.2 ± 11.6	0.72
Male gender, n (%)	76 (81)	14 (70)	0.34
Body mass index, kg/m^2^	28.7 ± 4.3	–	–
Duration of diabetes, years	5.1 ± 4.5	–	–
HbA1c, mmol/mol	61.6 ± 15.6	37.1 ± 4.6	< 0.0001
Median HbA1c since diagnosis, mmol/mol	63.8 ± 15.2	–	–
Maximum HbA1c since diagnosis, mmol/mol	85.0 ± 24.6	–	–
Microalbuminuria, n (%)	45 (48)	–	–
Systolic BP, mmHg^a^	131.1 ± 15.2	127.8 ± 15.5	0.93
Diastolic BP, mmHg^a^	72.3 ± 9.0	76.9 ± 10.3	0.05
Total cholesterol, mmol/L	4.3 ± 1.1	5.2 ± 0.9	0.003
Smoking	15 (16)	0	–
Metformin	83 (88)	0	–
Sulphonylurea	32 (34)	0	–
Gliptin	10 (11)	0	–
Other hypoglycaemic	4 (4)	0	–
Insulin	0	0	–
ACE inhibitor	0	0	–
Beta blocker	3 (3)	0	–
Calcium channel blocker	10 (11)	1 (5)	0.44
Diuretic	4 (4)	0	–
Statin	66 (70)	3 (15)	< 0.0001
Aspirin	16 (17)	2 (10)	0.43
Serum aldosterone, pmol/L	306.3 ± 18.8	–	–
High sensitivity C reactive protein, mg/L	3.5 ± 5.5	–	–
Ascending aortic distensibility, 10^−3^ mmHg^−1^	1.81 ± 1.16	2.78 ± 1.67	0.002
Descending aortic distensibility, 10^−3^ mmHg^−1^	2.11 ± 1.05	3.47 ± 1.54	0.0002
Pulse wave velocity, m/s	8.00 ± 2.87	7.58 ± 2.11	0.74

*ACE* angiotensin converting enzyme, *CVD* cardiovascular disease

^a^24 h blood pressure in patients with diabetes mellitus, clinic blood pressure in controls

Baseline AAD was 1.81 ± 1.16 10^−3^ mmHg^−1^, DAD was 2.11 ± 1.05 10^−3^ mmHg^−1^ and PWV was 8.00 ± 2.87 m/s. In healthy controls AAD was 2.78 ± 1.67 10^−3^ mmHg^−1^, DAD was 3.47 ± 1.54 10^−3^ mmHg^−1^ and PWV was 7.58 ± 2.11 m/s. AAD and DAD were significantly lower in patients with diabetes than matched controls (P = 0.002 and 0.0002 respectively). The difference in PWV was not significant (P = 0.74).

### Association between demographic and risk factors and aortic parameters

Ascending aortic distensibility had significant associations with age (Beta − 0.063, P < 0.0001), current HbA1c (Beta − 0.016, P = 0.04), maximum HbA1c since diagnosis (Beta − 0.011, P = 0.02), sulphonylurea use (Beta − 0.57, P = 0.02) and 24 h systolic blood pressure (Beta − 0.026, P = 0.001), Table 2. On multivariable linear regression associations with age, current HbA1c and 24 h systolic BP remained significant: Age (Beta − 0.068, P < 0.0001), current HbA1c (Beta − 0.017, P = 0.007) and 24 h systolic BP (Beta − 0.014, P = 0.03). In this regression model maximum HbA1c was of borderline significance (Beta − 0.0077, P = 0.05) but sulphonylurea use was no longer significant (Beta − 0.12, P = 0.53).

**Table 2:** Linear regression of association between aortic stiffness and clinical factors with significant associations in italic

	AAD			DAD			PWV		
	Beta	95% CI	P value	Beta	95% CI	P value	Beta	95% CI	P value
Age	− *0.063*	− *0.080*; *−* *0.046*	< *0.0001*	*−* *0.052*	*−* *0.068; 0.036*	*<* *0.0001*	*0.11*	*0.064; 0.16*	*<* *0.0001*
Male gender	0.13	− 0.47; 0.73	0.69	− 0.18	− 0.73; 0.36	0.50	0.11	− 1.39; 1.61	0.88
Body mass index	0.0042	− 0.051; 0.060	0.88	− 0.030	− 0.080; 0.020	0.24	− 0.30	− 0.17; 0.11	0.67
Duration of diabetes	− 0.037	− 0.090; 0.016	0.17	− 0.040	− 0.090; 0.0050	0.08	0.087	− 0.044; 0.22	0.19
*HbA1c*	*−* *0.016*	*−* *0.031*; *−* *0.00073*	*0.04*	− 0.00037	− 0.014; 0.013	0.96	0.027	− 0.010; 0.065	0.15
Median HbA1c since diagnosis	− 0.013	− 0.029; 0.0026	0.10	0.00084	− 0.013; 0.015	0.91	0.019	− 0.020; 0.058	0.33
Maximum HbA1c since diagnosis	*−* *0.011*	*−* *0.020*; *−* *0.0014*	*0.02*	− 0.0035	− 0.012; 0.0050	0.42	0.0083	− 0.016; 0.032	0.50
Microalbuminuria	− 0.21	− 0.69; 0.26	0.37	− 0.21	− 0.64; 0.22	0.34	0.89	− 0.28; 2.06	0.13
24 h systolic BP	*−* *0.026*	*−* *0.040*; *−* *0.011*	*0.001*	*−* *0.020*	*−* *0.034; −* *0.0067*	*0.004*	*0.040*	*0.0014; 0.078*	*0.04*
24 h diastolic BP	− 0.0058	− 0.033; 0.021	0.67	− 0.0027	− 0.027; − 0.022	0.83	− 0.0015	− 0.068; 0.065	0.97
Total cholesterol	0.15	− 0.062; 0.36	0.16	0.056	− 0.14; 0.25	0.57	− 0.44	− 0.97; 0.081	0.10
Smoking	0.11	− 0.54; 0.76	0.74	0.098	− 0.49; 0.69	0.74	− 0.59	− 2.20; 1.02	0.47
Metformin	0.27	− 0.47; 1.01	0.47	0.35	− 0.31; 1.02	0.30	0.32	− 2.16; 1.51	0.73
Sulphonylurea	− *0.57*	− *1.06; −* *0.08*	*0.02*	0.34	− 0.79; 0.11	0.14	1.17	− 0.056; 2.39	0.06
Gliptin	− 0.31	− 1.08; 0.46	0.43	0.14	− 0.57; 0.83	0.70	0.73	− 1.18; 2.64	0.45
Statin	− 0.048	− 0.57; 0.47	0.86	− 0.13	− 0.60; 0.35	0.60	0.38	− 0.91; 1.67	0.56
Aspirin	− 0.61	− 1.23; 0.012	0.06	− 0.51	− 1.08; − 0.51	0.07	1.10	− 0.46; 2.66	0.16

Significant associations in italics

Abbreviations as in Table 1

DAD had significant associations with age (Beta − 0.052, P < 0.0001) and 24 h systolic BP (Beta − 0.020, P = 0.004) but no indices related to diabetes. PWV had significant associations with age (Beta 0.11, P < 0.0001) and 24 h systolic BP (Beta 0.040, P = 0.04) but no indices related to diabetes.

### Cardiovascular disease events

Patients were followed up for 882 ± 146 days. 19 patients (20%) had a CVD event including silent MI on baseline scan 15 (16%), stroke 3 (3%), cardiovascular death 2 (2%), ST elevation MI 2 (2%), silent MI on follow up scan 1 (1%), percutaneous coronary intervention 2 (2%), heart failure 1 (1%) and arrhythmia 1 (1%). 9/19 subjects with CVD events were asymptomatic and were only detected on CMR. 1 patient died from non-cardiovascular causes (malignancy).

The differences in aortic stiffness between those with silent MI on baseline scan (N = 15) and without silent MI did not reach statistical significance (AAD 1.33 ± 0.89 vs 1.90 ± 1.19 10^−3^ mmHg^−1^, P = 0.0.8; DAD 1.76 ± 1.13 vs 2.18 ± 1.03 10^−3^ mmHg^−1^ P = 0.12; PWV 8.31 ± 2.14 vs 7.94 ± 3.00 m/s).

On logistic regression of the aortic parameters only AAD had a significant association with CVD events; hazard ratio (HR) 0.49, 95% confidence interval (CI) 0.25–0.95, P = 0.01, Table 3. The associations of DAD and PWV with CVD events were not significant (P = 0.19 and 0.45 respectively). Smoking was the only individual risk factor to have a trend to association with CVD events (HR 3.38, 95% CI 1.03–11.15, P = 0.05).

**Table 3:** Logistic regression of the association between aortic stiffness and clinical factors with CVD events

	Hazard ratio	95% CI	P value
AAD	*0.49*	(*0.25*; *0.95*)	*0.01*
DAD	0.70	(0.41; 1.20)	0.19
PWV	1.07	(0.90; 1.26)	0.45
Age	1.05	(1.00; 1.10)	0.07
Gender	0.19	(0.02; 1.52)	0.12
Body mass index	0.97	(0.86; 1.09)	0.62
Duration of diabetes	0.99	(0.88; 1.10)	0.80
HbA1c	0.98	(0.95; 1.02)	0.35
Median HbA1c since diagnosis	1.01	(0.97; 1.04)	0.75
Maximum HbA1c since diagnosis	1.00	(0.97; 1.02)	0.74
Microalbuminuria	2.19	(0.77; 6.16)	0.13
24 h systolic BP	1.02	(0.99; 1.06)	0.18
24 h diastolic BP	1.01	(0.95; 1.06)	0.80
Total cholesterol	0.89	(0.56; 1.43)	0.64
Smoking	*3.38*	(*1.03*; *11.15*)	*0.05*
Serum aldosterone	1.00	(0.99; 1.00)	0.33
High sensitivity C reactive protein	0.99	(0.90; 1.09)	0.87

Significant associations in italics

*CI* confidence interval, *AAD* ascending aorta distensibility, *DAD* descending aorta distensibility, *PWV* pulse wave velocity, *CVD* cardiovascular disease

### Response of aortic stiffness to RAAS inhibition

25 patients with persistent microalbuminuria were treated with RAAS inhibition and had a repeat CMR 365 ± 38 days after the initial scan. Prescribed RAAS inhibition included ramipril 19, losartan 3, perindopril 1, candesartan 1, irbesartan 1 equivalent to a dose of ramipril 4.9 ± 3.1 mg. RAAS inhibition was associated with a non-significant decrease in blood pressure of 5 ± 16 mmHg in systolic and 3 ± 8 mmHg in diastolic blood pressures. Over follow up there was no significant change in weight (86.2 ± 11.1 to 86.7 ± 11.5 kg, P = 0.51) or HbA1c (60.1 ± 17.7 to 61.8 ± 14.7 mmol/mol, P = 0.57). Treatment with RAAS inhibition was associated with a significant increase in AAD of 0.47 ± 1.04 10^−3^ mmHg^−1^ but no significant change in DAD or PWV (P = 0.92 and 0.42 respectively). After treatment with RAAS inhibition AAD was increased but was still significantly lower than in healthy controls (1.97 ± 1.07 10^−3^ mmHg^−1^ vs 2.78 ± 1.67 10^−3^ mmHg^−1^, P = 0.04) (Table 4).

**Table 4:** Baseline characteristics and change in AAD, DAD and PWV after 1 year treatment with RAAS inhibition in 25 subjects

	Baseline	Follow up	P value
Age	64.2 ± 11.8		
Male gender, N (%)	22 (88%)		
Body mass index, kg/m^2^	29.1 ± 3.4		
Duration of diabetes, years	5.2 ± 4.5		
HbA1c, %	7.6 ± 1.5		
HbA1c, mmol/mol	60.1 ± 17.7		
Median HbA1c since diagnosis, mmol/mol	63.4 ± 17.6		
Maximum HbA1c since diagnosis, mmol/mol	86.6 ± 25.6		
24 h systolic BP, mmHg	136.7 ± 19.3	131.6 ± 20.9	0.12
24 h diastolic BP, mmHg	72.9 ± 10.1	70.7 ± 11.1	0.07
Total cholesterol, mmol/L	4.1 ± 1.0		
Smoking, N (%)	4 (16%)		
Serum aldosterone, pmol/L	337.0 ± 190.8	238.8 ± 138.2	0.11
AAD (10^−3^ mmHg^−1^)	*1.51 *±* 1.15*	*1.97 *±* 1.07*	*0*.*007*
DAD (10^−3^ mmHg^−1^)	1.98 ± 1.29	1.96 ± 1.10	0.92
PWV (m/s)	8.95 ± 2.60	8.33 ± 3.58	0.42

Significant associations in italics

Abbreviations as in Table 1

## Discussion

We have demonstrated increased aortic stiffness by CMR in asymptomatic patients with type 2 diabetes compared to healthy controls. We have also shown that AAD, DAD and PWV are significantly influenced by age and 24 h systolic BP. However only AAD had an association with HbA1c, which remained significant after correction for age and BP. AAD had a significant association with CVD events over 2.4 years follow up. Furthermore, no other traditional cardiovascular risk factors or marker of glycaemic control had an association with CVD events. Finally, we have shown with RAAS inhibition that AAD improves towards that of healthy controls.

The findings that AAD is associated with glycaemic control, adverse CVD events and that it is improved on treatment suggest that AAD has a potential role as an imaging marker of cardiovascular risk in asymptomatic patients with type 2 diabetes, although the cost and availability of CMR may be prohibitive for routine clinical use.

### AAD and glycaemia

In the present study AAD was the only parameter of aortic stiffness to have a significant association with glycaemic control, independent of blood pressure. Previous studies have shown an association between dysglycaemia and PWV [25, 26] but previous CMR studies showing that diabetes is associated with decreased AAD were either not powered to show an association with glycaemic control [4, 5, 27] or data on glycaemic agents and glycaemic control were not reported [6]. The association of AAD with sulphonylurea therapy is potentially interesting as it may implicate hypoglycaemia in the observed changes in AAD. However, this relationship was not significant after correction for HbA1c suggesting it is merely a marker of worse glycaemic control. Taken together, glycaemic control appears to influence AAD and hence glucose levels may play a direct role in stiffening of the aorta. Preliminary data suggests that aortic stiffness may be reduced by the sodium-glucose co-transporter-2 inhibitor dapagliflozin [28] and larger studies are required to confirm whether this is mediated by glucose lowering or other mechanisms.

### AAD and CVD events

In the present study decreasing AAD appeared to be a marker of cardiovascular disease events, independently of conventional risk factors. The association between AAD and outcome in patients with diabetes are in agreement with previous studies demonstrating the prognostic importance of AAD measured by CMR in asymptomatic cohorts of patients of varied cardiovascular risk (including a minority with diabetes). AAD was measured in 3675 subjects from the Multi-Ethnic Study of Atherosclerosis study who were followed up for 8.5 years [11]. In this period decreased AAD was associated with increased all-cause mortality and CVD events (myocardial infarction, stroke, cardiac arrest and cardiovascular death). This risk was independent of conventional risk factors. In the Dallas Heart Study, both AAD and PWV were measured using CMR in 2122 participants free from cardiovascular disease [12]. After correction for traditional risk factors AAD and PWV had weak associations with nonfatal cardiac events and nonfatal extra-cardiac events but not cardiovascular death.

The prognostic importance of aortic PWV has been extensively studied by applanation tonometry with clear evidence of an incremental benefit over traditional risk factors for the prediction of cardiovascular events [29]. This technique has been used specifically in patients with diabetes and has shown that PWV is increased in diabetes independently of BP and associated with increased mortality [30]. PWV was not associated with CVD events in the present study and may reflect measurement of flow in a shorter section of aorta (arch only in our study compared with carotid to distal abdominal aorta by tonometry) or significantly worse temporal resolution than with tonometry.

### Reversing AAD

A subgroup of 25 patients in the present study were treated with RAAS inhibition for newly diagnosed microalbuminuria. In these patients RAAS inhibition was associated with a significant increase in AAD (but not DAD or PWV). It is well recognised that patients with albuminuria have markedly elevated cardiovascular risk [13, 14] which may in part be reduced by RAAS inhibition [15, 16]. Although only an observational finding we believe that ours is the first study to show that aortic stiffness associated with diabetes can be reduced by medical intervention. A previous randomised study has shown that RAAS modulation with spironolactone can decrease aortic stiffness in patients with chronic kidney disease [17]. Our findings suggest the same might be true in diabetes although larger randomised trials are needed to establish whether decreasing aortic stiffness by RAAS inhibition leads to improved outcomes.

Recent trials of hypoglycaemic agents in type 2 diabetes have included patients with established CVD [31] or at risk of CVD [32] and have shown that it is possible to improve CVD outcomes in these patient groups. AAD measured by CMR has the potential to be used as a surrogate endpoint in future studies of hypoglycaemic agents to identify those with increased CVD risk who are most likely to demonstrate mortality benefit. Furthermore AAD could easily be added to a scan protocol including comprehensive assessment of cardiac structure function and tissue characteristics.

## Limitations

This was an observational non-randomised study and the interventional component needs to be repeated in a larger randomised study, although it can be argued that this is a strength as patient selection was limited and they were studied under real life, and not randomised controlled trial, conditions. 9/19 patients with CVD events were asymptomatic with MI being detected on LGE CMR. However in patients with diabetes the mortality associated with unrecognised MI is significant and is comparable to those with clinically recognised MI [33, 34]. The temporal resolution of the CMR PWV was at least 10 times lower than that achieved by tonometry and the lack of association between PWV and glycaemia or CVD events may reflect a limitation of the technique used. There was a male preponderance in this cohort reflecting the different cut-offs for ACR, which may have influenced our findings. The blood pressure measurement for AD calculation was performed peripherally which due to the pressure amplification phenomenon could lead to overestimation of stiffness, although the identical protocol was used for all subjects therefore minimising bias.

## Conclusions

Ascending aortic distensibility measured by CMR is independently associated with poor glycaemic control and adverse cardiovascular events. Furthermore, it appears to be reversible on treatment with RAAS inhibition. AAD is a promising marker of cardiovascular risk in asymptomatic patients with type 2 diabetes and has potential use as a surrogate cardiovascular endpoint in studies of novel hypoglycaemic agents.

## Abbreviations

AAD: ascending aortic distensibility
ACE: angiotensin converting enzyme
ARB: angiotensin receptor blocker
BP: blood pressure
CMR: cardiovascular magnetic resonance
CV: coefficient of variability
CVD: cardiovascular disease
DAD: descending aortic distensibility
LGE: late gadolinium enhancement
PWV: pulse wave velocity
RAAS: renin–angiotensin–aldosterone system
